# Knowledge-based planning for multi-isocenter VMAT total marrow irradiation

**DOI:** 10.3389/fonc.2022.942685

**Published:** 2022-10-04

**Authors:** Kang-Hyun Ahn, Damiano Rondelli, Matthew Koshy, Julien A. Partouche, Yasmin Hasan, Hongtao Liu, Kamil Yenice, Bulent Aydogan

**Affiliations:** ^1^ Department of Radiation Oncology, University of Illinois, Chicago, IL, United States; ^2^ Department of Radiation and Cellular Oncology, University of Chicago, Chicago, IL, United States; ^3^ Division of Hematology/Oncology, University of Illinois, Chicago, IL, United States

**Keywords:** knowledge-based planning, RapidPlan, VMAT, total marrow irradiation, TMI, radiotherapy

## Abstract

**Purpose:**

Total marrow irradiation (TMI) involves optimization of extremely large target volumes and requires extensive clinical experience and time for both treatment planning and delivery. Although volumetric modulated arc therapy (VMAT) achieves substantial reduction in treatment delivery time, planning process still presents a challenge due to use of multiple isocenters and multiple overlapping arcs. We developed and evaluated a knowledge-based planning (KBP) model for VMAT-TMI to address these clinical challenges.

**Methods:**

Fifty-one patients previously treated in our clinic were selected for the model training, while 22 patients from another clinic were used as a test set. All plans used a 3-isocenter to cover sub-target volumes of head and neck (HN), chest, and pelvis. Chest plan was performed first and then used as the base dose for both the HN and pelvis plans to reduce hot spots around the field junctions. This resulted in a wide range of dose-volume histograms (DVH). To address this, plans without the base-dose plan were optimized and added to the library to train the model.

**Results:**

KBP achieved our clinical goals (95% of PTV receives 100% of Rx) in a single day, which used to take 4-6 days of effort without KBP. Statistically significant reductions with KBP were observed in the mean dose values to brain, lungs, oral cavity and lenses. KBP substantially improved 105% dose spillage (14.1% ± 2.4% vs 31.8% ± 3.8%), conformity index (1.51 ± 0.06 vs 1.81 ± 0.12) and homogeneity index (1.25 ± 0.02 vs 1.33 ± 0.03).

**Conclusions:**

KBP improved dosimetric performance with uniform quality. It reduced dependence on planner experience and achieved a factor of 5 reduction in planning time to produce quality plans to allow its wide-spread clinical implementation.

## 1 Introduction

Allogeneic stem cell transplantation commonly requires total body irradiation (TBI) to provide a sufficient level of immunosuppression in addition to killing malignant cells (1–3). Although higher radiation dose would improve disease-free survival, injuries to critical organs compel the radiation to be targeted more selectively (4, 5). Over the past decade, radiation dose sculpting to the total marrow has been implemented to substantially decrease dose to normal organs using techniques of intensity modulated radiation therapy (IMRT) (6, 7) and helical tomotherapy (8, 9). In particular, recent studies of volumetric modulated arc therapy (VMAT) total marrow irradiation (TMI) demonstrated satisfactory plan quality and treatment delivery efficiency (10–13). Recent efforts enabled clinical implementation of TMI through several Phase I and II clinical trials for advanced acute myeloid leukemia and multiple myeloma patients proving its clinical feasibility and tolerability with encouraging outcome results (14–17). There is now worldwide interest for the implementation of TMI especially for those patients with advanced diseases who could benefit from intensified treatment regiments.

Treatment planning for TMI, however, is an extremely resource-intensive procedure as the inverse planning optimization requires extended computing time and planner interventions to work out a plan for the huge target volumes encompassing total marrow. These difficulties, along with variations in knowledge and experience, can lead to inconsistent treatment plan qualities and remain the major hurdle for widespread clinical application of TMI.

Knowledge-based radiotherapy treatment planning (KBP), in which new plan dose volume histograms (DVH) and optimization objectives are predicted from libraries of the historical plan data (18–22), suggests a possibility to expedite the demanding VMAT-TMI planning with reduced iterations of planner-intervention during optimization. Commercially available KBP solutions analyze the field geometry, patient anatomy and DVH’s of the past plans to train a model using principal component analysis and generates patient-specific achievable plan objectives along with estimated DVH’s for organs at risk (OARs) (23–25).

Although KBP approach has been investigated in various anatomical sites and proved its value to facilitate challenging treatment planning processes with consistent plan quality, TMI adds an extra difficulty for KBP application as the large target volume requires multiple isocenters plans and a base-dose optimization technique, which compound the model training. In this study, we developed a KBP model to handle multiple-isocenter VMAT TMI and assessed its performance and robustness using patient data from two clinics.

## 2 Methods and materials

### 2.1 Patient selection

Fifty-one patients previously treated at the University of Illinois at Chicago (UIC) from 2009 to 2020 were selected for the model training, and 22 patients treated at the University of Chicago (UChicago) were selected to test the model. All patients in this study were treated under clinical trials approved by their respective institutional review boards (IRB). Target and critical structures were contoured and planned using the Eclipse treatment planning system and RapidPlan version 15.6 (Varian Medical Systems, Palo Alto, CA). Although the patients had been contoured in the consistent way, we observed a gradual improvement in plan quality - as defined in the Evaluation section - with the upgrade of Varian optimization software over the past decade to exploit advanced techniques and user interface such as Photon Optimizer algorithm (26, 27) and Arc Geometry tool (28). To minimize the dosimetric performance variation in the reference set for the evaluation of our KBP, we identified a benchmark cohort of recent 11 clinically approved plans optimized by a single planner at UIC with similar target volumes. The average planning target volume (PTV) sizes were 7470 ± 880 cc (range: 6383 - 9389 cc) and 7770 ± 1190 cc (range: 5702 - 9489 cc) for the 11 clinical and 22 test patients, respectively.

### 2.2 VMAT-TMI plan preparation

For all patients, clinical target volume (CTV) was defined as bones from head to mid-femur and was contoured in the whole-body simulation computed tomography (CT) of 3-mm slice thickness. PTV was generated by adding a 3-mm margin around the CTV and was divided into three sub volumes to form separate targets for head and neck (H&N), chest, and the pelvis. Our institutional practice has been to exclude extremities and mandible from the PTV due to treatment field length limitation and acute toxicities in the oral cavity, respectively. Further justification for the exclusion of extremities is the fact that there is no active bone marrow in adults. Nevertheless, we recently started including mandible after a local recurrence case (29). Also, a careful field junction setup can achieve optimal target coverage including legs (30). However, for a consistent evaluation with the benchmark cohort of clinically approved plans, we maintained the same target volume definition in this study to exclude extremity bones below mid-humerus in arms and below mid-femur in legs, mandible and maxillary structures from the PTV. Three-isocenter plans with 13 arcs delivered 3 Gy dose in two fractions per day. H&N and pelvis plans were optimized utilizing the chest plan as the base dose to prevent hot spots around the field junctions. Chest plans used five 280° arcs, and both H&N and pelvis plans used four 280° arcs each to have all area of target well visible by the jaw opening. The Eclipse Arc Geometry tool reported the point with the lowest coverage was seen by at least 30% of the control points. Each arc had an upper jaw (y-axis) opening of 40-cm and a lower jaw (x-axis) opening of 15-cm to achieve full modulation performance of the Millenium™ MLC. Optimization goals were set to cover 95% of PTVs by the prescription dose with up to 140% hot spots allowed within the targets. All plans used 6-MV beams and Anisotropic Analytical Algorithm as dose calculation method with 2.5-mm dose resolution grid.

### 2.3 Model training

Three model libraries were created separately for H&N, chest and pelvis using 51 training plans. In an ideal data-rich environment, the patient data set needs to be randomly divided into a training set, a validation set, and a test set. The validation step can estimate prediction error for model selection before the test set assesses the generalization error of the final model. However, with the slow accumulation of patient data for TMI (51 patients in one center over the past decade), we adopted a continuous validation approach to maximally exploit the limited number of patients. If plan qualities of the training patients got improved with KBP, the improved plan was added back to the same model to continuously improve the quality of the model. The final model thus created was assessed using the test set of 22 UChicago patients that was unknown to the model. Each model was designed to generate estimated mean dose values of OARs with priority values set to the institutional practice. With the relative priority values of 100 assigned for PTV coverage, the mean dose constraints priorities were set 60 for the brain and lungs, and 30 or 40 for all other critical organs.

**Figure 1:**
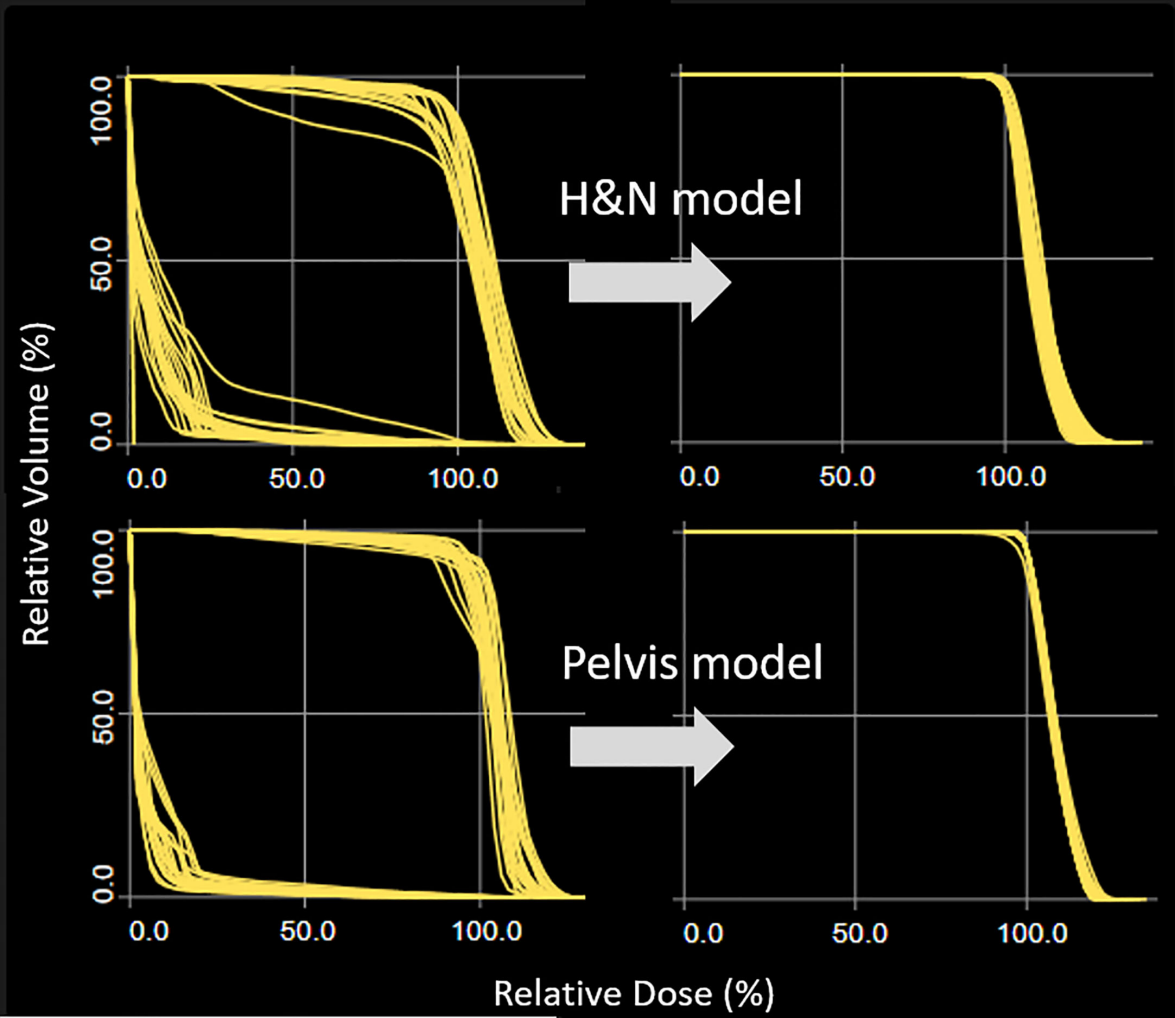
Target DVH distribution for H&N and pelvis plans. Optimization when chest plan is used as the base dose resulted in a wide target DVH range (left column). The scattered DVH distribution was successfully removed once the model plans were re-optimized without the base dose.

The H&N and pelvis plans optimized with base dose resulted in a wide range of DVH as shown in **Figure 1** (left). The scattered distribution due to chest base dose distribution was successfully removed once the model plans were re-optimized without the base dose. These re-optimized plans would generate hot spots at field junctions in the plan sum and were not clinically deliverable. They served as KBP model plans to drive better DVH estimation for H&N and pelvis (**Figure 1**, right).

### 2.4 DVH estimation and optimization

The KBP model trained using 51 UIC patients was used to generate DVH estimates and dose objectives for 3 sub plans for each of the 22 test patients. In addition to these KBP objectives, we created 1-cm thick ring structures around PTV to improve dose conformity near the target volume and turned-on normal tissue objective with 1-cm distance from the target border to further suppress dose outside the ring structures. Eclipse optimization conveniently displays the calculation cost per structure to enable modifying the relative computing effort on the fly. The relative priority values of the ring structure and the normal tissue objective were intermittently adjusted during the optimization to maintain these calculation costs comparable to that of the target volume.

For H&N and pelvis plans, the training model predictions of OARs depended on their location relative to the junctions. For brain or eyes, for instance, the predicted DVHs would be adequate to guide the base-dose optimization - even though the H&N model ignored the dose contribution from the chest plan - because the OARs are away from the junctions and the dose contribution from the chest plan would be negligible. For kidneys or bowel, on the other hand, the predicted DVHs from pelvis plan would substantially underestimate the final dose because the model could not account for the base dose contribution from the chest plan. Nevertheless, the sums of predicted mean dose values from chest and pelvis plans would be the upper limits of the composite mean dose values, and were used as the surrogate optimization objectives. Note that a composite DVH cannot be calculated from two subplan DVHs, but a composite mean dose can be calculated from the corresponding subplan DVHs by simply adding the respective mean values (see the **Supplemental Figure**).

### 2.5 Evaluation

KBP performance on 22 test patients independent of the training set (open-loop) was benchmarked using 11 patients treated between 2018 and 2019. Also, one of those 11 patients was selected to run closed-loop KBP to demonstrate its performance on the same patient CT. Plan quality was evaluated using homogeneity index (D0.03cc/D100%), conformity index (V95%/PTV), dose spillage (V105%-PTV)/PTV), and total MU’s. Student t-tests (two-tails, two-sample unequal variance) identified significant differences in mean dose values of PTVs and OARs. F-tests evaluated if two variances were significantly different.

## 3 Results

**Figure 2:**
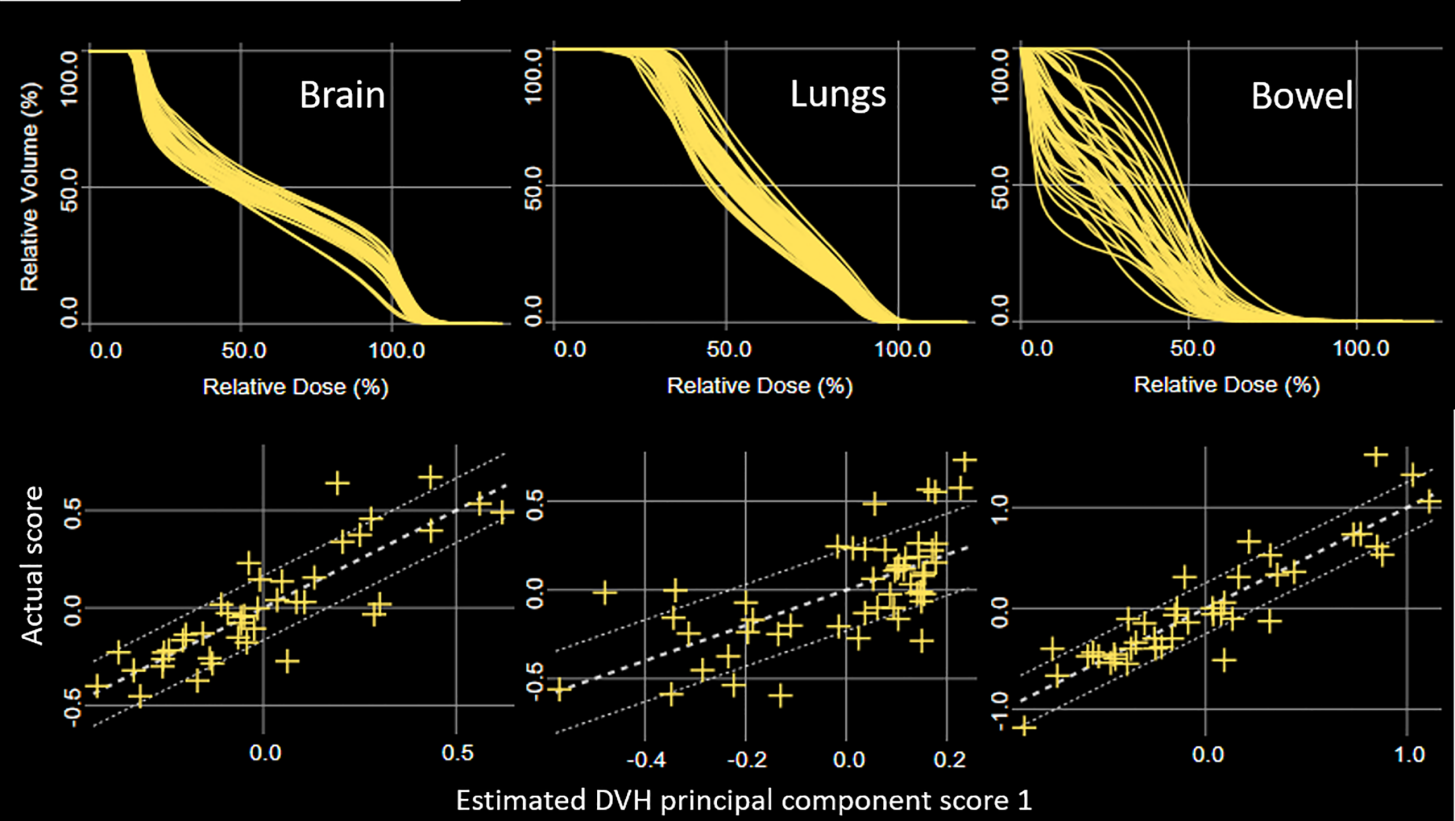
DVH plots (top) and principal component scatter plots (bottom) for brain, lungs, and bowel in the HN, chest, and pelvis models, respectively. Confidence intervals are displayed using one standard deviation of fitting error (bottom).

Model training statistics for the 51 patients are summarized in **Table 1**. Note that lungs overlap with both H&N and chest models. Liver, bowel, and kidneys overlap with both chest and pelvis models. The coefficients of determination (R^2^) were in the range 0.40 (lungs, chest model) – 0.96 (liver, pelvis model), and chi squares were in the range 1.04 (lungs, chest model) – 1.17 (oral cavity, H&N model). The training model detected potential outliers using all of the statistics for each structure and reported substantially large number of outliers for kidneys in the chest model and bowel in the pelvis model. **Figure 2** shows DVH plots and residual plots for brain, lungs, and bowel in the H&N, chest, and pelvis models, respectively. The residual plots evaluate how the original DVH differs from the estimated DVH by showing the first principal component scores of the actual and estimated DVH for each structure.

**Table 1:** Summary of model training.

Model	Structure	Coeff. of Determination (R^2^)	Chi Square	Outliers/Matched Structures
H&N	brain	0.701	1.164	11/41
	eyes	0.535	1.077	12/41
	lenses	0.615	1.077	7/41
	oral cavity	0.743	1.170	10/41
	lungs	0.955	1.047	1/41
chest	lungs	0.402	1.040	5/51
	heart	0.681	1.101	5/51
	liver	0.689	1.122	4/51
	kidneys	0.503	1.069	24/51
	bowel	0.924	1.111	13/51
pelvis	liver	0.956	1.096	7/41
	kidneys	0.852	1.053	2/41
	bowel	0.770	1.108	20/41

The KBP models generated achievable mean dose values along with the prediction ranges of DVHs for each OAR for the 22 test patients. The estimated objectives were attained within a single day using 2~3 iterative optimizations, which used to take 4-6 days of effort for clinical plans without using the knowledge-based approach. **Table 2** shows a comparison of mean dose values to organs and targets for the KBP test plans and benchmark clinical plans. P-values are shown in bold if differences are statistically significant (<0.05). Overall KBP had comparable dosimetric results as clinical plans and achieved significant improvement in major OARs. The mean dose values (% of prescribed dose) for brain and lungs decreased from 58.5% ± 3.9% to 54.5% ± 2.5%, and from 64.1% ± 3.3% to 60.3% ± 1.9%, respectively. Although both groups were normalized to cover 95% of PTV’s with the prescription dose, KBP achieved the coverage with lower mean dose to the chest and H&N target volumes. In addition, KBP had narrower distribution of mean dose values for lungs and each of the PTVs as indicated by p-values of F-tests.

**Table 2:** Comparison of mean dose values of OAR (% of prescribed dose).

	Clinical plans	KBP	P-value	
			Average (T-test)	St.Dev. (F-test)
Brain	58.5 ± 3.9	54.5 ± 2.5	**0.008**	0.08
Heart	51.5 ± 3.6	50.9 ± 3.4	0.77	0.81
Lungs	64.1 ± 3.3	60.3 ± 1.9	**0.002**	**0.03**
Bowel	47.7 ± 5.8	46.5 ± 5.0	0.95	0.54
Liver	52.7 ± 4.5	54.0 ± 2.9	0.32	0.10
Kidneys	49.7 ± 5.5	50.5 ± 4.9	0.37	0.63
Eyes	44.2 ± 3.3	41.6 ± 2.6	0.07	0.35
Oral Cavity	34.7 ± 4.1	27.0 ± 3.2	**<0.001**	0.33
Lenses	29.4 ± 1.9	25.9 ± 1.8	**<0.001**	0.80
PTV chest	111.7 ± 1.5	108.5 ± 0.5	**<0.001**	**<0.001**
PTV HN	111.0 ± 1.6	109.0 ± 0.5	**0.002**	**<0.001**
PTV Pelvis	108.0 ± 0.9	108.0 ± 0.5	0.77	**0.03**
Body	56.8 ± 3.9	57.0 ± 5.1	0.44	0.36

Statistically significant p-values (<0.05) are in bold.

KBP also had better plan qualities as shown in **Table 3**. Average homogeneity index (D0.03cc/D100%) and conformity index (V95%/PTV) decreased from 1.33 ± 0.03 to 1.25 ± 0.02, and from 1.81 ± 0.12 to 1.51 ± 0.06, respectively. Note that standard deviation of the two indices also significantly decreased as confirmed by F-tests. Furthermore, KBP achieved a factor of two reduction in the 105% dose spillage by decreasing it from 31.8% ± 3.8% to 14.1% ± 2.4%. The slight increase in total MU’s for KBP was statistically insignificant (p-value = 0.08).

**Table 3:** Comparison of plan quality.

	Clinical plans	KBP	P-value	
			Average (T-test)	St.Dev. (F-test)
D0.03cc	1.33 ± 0.03	1.25 ± 0.02	**<0.001**	**0.03**
V95%/PTV	1.81 ± 0.12	1.51 ± 0.06	**<0.001**	**0.01**
Dose spillage	31.8 ± 3.8	14.1 ± 2.4	**<0.001**	0.06
Total MU	3245 ± 387	3492 ± 265	0.08	0.14

Statistically significant p-values (<0.05) are in bold.

**Figure 3:**
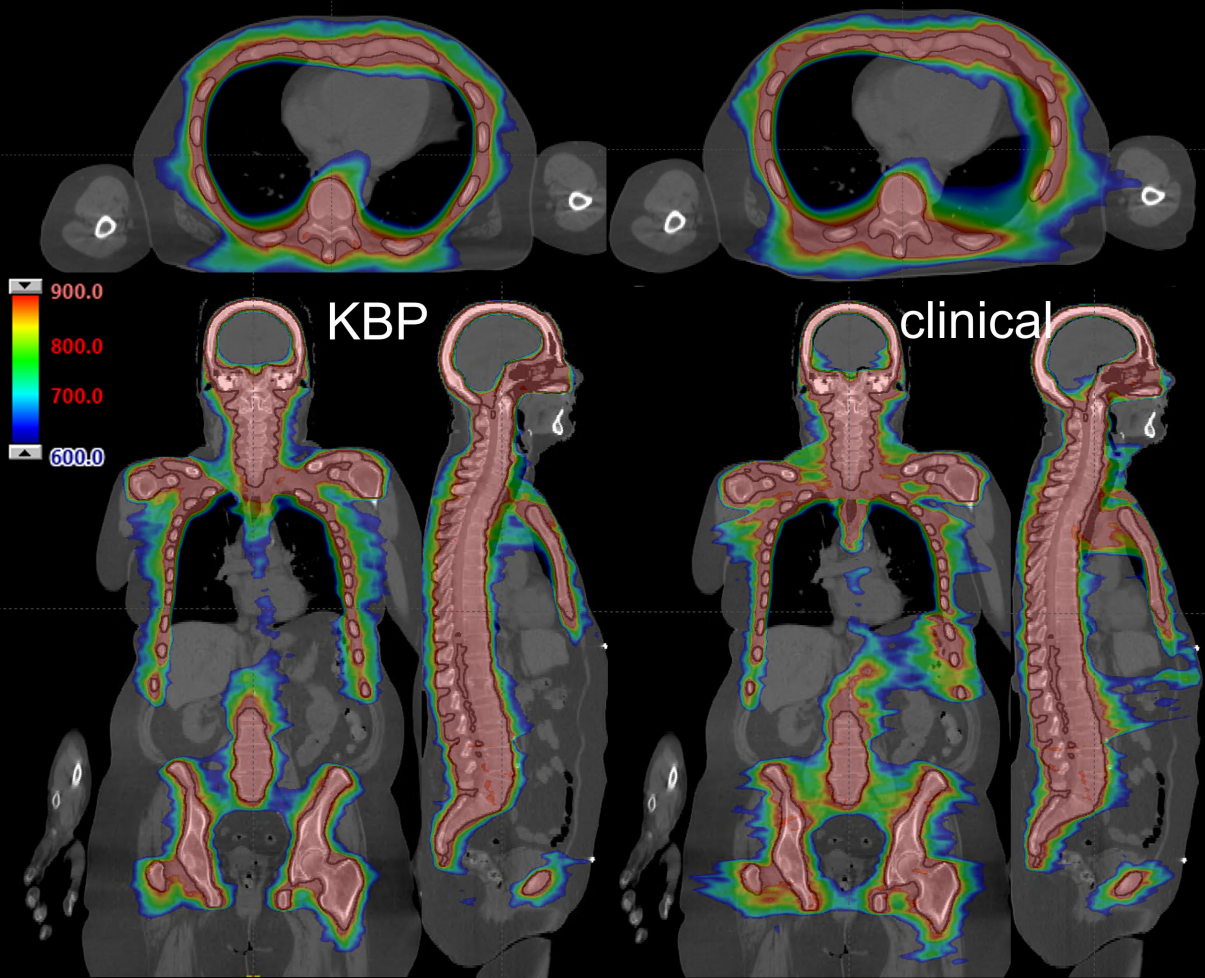
An example of KBP performance (left) compared to a clinical plan (right). Black lines depict PTV. Dose range shown from 900 cGy (red) to 600 cGy (blue) in axial (top), coronal (bottom left), and sagittal plane (bottom right).

**Figure 3** shows an example of KBP performance (left) compared to a clinical plan (right) with axial, coronal, and sagittal views. As there was no overlapping between the test set and the benchmark clinical set, a closed-loop KBP validation was performed on one patient selected from the clinical set for a fair comparison on the same patient CT. Both plans had acceptable mean dose values for OARs close to those reported in **Table 2**. For instance, mean brain and lung dose values were 58% and 61% for the KBP and 63% and 62% for the clinical plan, respectively. The homogeneity index, conformity index, dose spillage, and total MU were 1.27, 1.62, 20.8%, and 3634 for the KBP, and 1.32, 1.95, 35.6%, and 3507 for the clinical plan, respectively. Overall, the color-wash dose distribution shown in **Figure 3** demonstrates a visible improvement in dose conformity indicated by the 600 cGy dose level.

## 4 Discussion

Linac-based multiple-isocenter TMI planning used base-dose optimization, and the model configuration was performed using re-optimization. The plots for brain and bowel in **Figure 2** were generated from the models of re-optimized plans with base-dose turned off. The results, or predicted objectives, could not be used for TMI directly, and we had to go through a few more steps for OARs at the field junctions as described in section 2.4. Our study is the first attempt to use RapidPlan for multiple adjacent plans with base-dose optimization, and we provide a working solution on how to handle the scattered target DVH distribution as shown in **Figure 1**. As indicated in **Table 1**, our approach enabled the model training with each of the OAR having sufficient number of instances for fitting the regression model. It should be noted that field overlapping of subplans inevitably resulted in OARs at the border partially covered by each subplan. In particular, kidneys and bowel were split by the chest and the pelvis subplans at arbitrary positions thus increased the number of outliers possibly due to the variability of in-field volume for the corresponding models. The goodness of fit statistics shown in **Table 1**, however, were supported by a favorable chi square (close to 1.0) and relatively robust to potential outliers, and KBP supported acceptable performance for all OARs as shown in **Table 2**. This also agrees with the previous studies that demonstrated robustness of RapidPlan for moderate proportions of dosimetric outliers (31, 32).

We note that KBP for general external beam cases employ substantially higher number of cases for training. However, there are reasonable differences in interpatient variation to support relatively small number of cases for TMI. For example, it is understandable that the H&N region KBP studies were performed with >100 patients with the wide variety of target shape (33, 34). The more variation treatment involves, the greater number of plans need to be included in the model. Every H&N cancer patient has different target shape, size, and the position of the target relative to OARs (far, close; overlapping, non-overlapping). On the other hand, every H&N TMI patient of ours had almost the same target shape, size and the geometrical relationships between the target and OARs.

Compared to typical coefficients of determination (R^2^, 0.7-0.9) in vendor-provided models for general clinical cases, **Table 1** reports low values for some structures. In particular, R^2^ for lungs in chest model was only 0.4, which could be due to the extremely complex target (rib cage) shape around the OAR, and would inevitably decrease the model prediction effect. Nevertheless, the chi square (1.040) indicates a reasonable performance of the fitting function and the mean lung dose predicted by KBP was achievable and even produced statistically significant improvement as shown in **Table 2**. This validates our approach and as a matter of fact is currently being used in at least two clinics with proven success. Again, it is worth noting that the convoluted target shape of TMI was repeated for each patient in a quite predictable way. Unlike general tumors where wide variety is observed for the target shape, size, and position, the PTV defined from bones presents relatively consistent target-OAR geometry for TMI, and this could be an advantageous aspect for model training with limited number of patients and the remarkably good results in this study.

To optimize the dose distribution of the large target from head to mid-femur, we developed three-iso, three-plan approach for TMI planning and implemented the KBP model to accommodate such configuration. While our current multi-plan approach breaks down a complex task of TMI into sub-problems to make them easier for the machine learning, it is inherently subject to increased number of dosimetric outliers at field overlapping area as discussed above and could potentially lead to suboptimal solution. Recent upgrade of Eclipse (version 16) opens up the possibility of an improvement by fully supporting both optimization and dose estimation of multiple isocenters in a single plan and calls for a further study that can potentially simplify the treatment delivery and improve plan quality as well.

One of the major difficulties for the inverse planning of large and complex target volumes is to figure out how far the planning can be pushed. Planners might end up either stopping the optimization prematurely or spending indefinite time in too many iterative optimizations without achieving clinically valid improvement. KBP provided attainable optimization goals ahead of treatment planning, and enabled substantial reduction in treatment planning time for the test patients which are not used for training the model. Moreover, **Table 2** shows that the knowledge-based optimization provided significantly decreased mean doses to brain, lungs, oral cavity and lenses even with the reduction of planning time. It is noteworthy that the improved therapeutic ratio is also implied by the decreased mean dose values to HN and chest PTV. For external beam therapy, the elevated mean target dose that achieves the same prescription coverage and conformity generally entails unnecessary radiation dose delivered to normal tissue.

Treatment planning is usually confronted with a trade-off problem among various dosimetric goals. Especially, pushing the optimization exceedingly to lower dose to OARs could compromise conformity, uniformity, or dose spillage to an unacceptable degree, or lead to increased beam modulation and complexity to deteriorate delivery accuracy along with increased time of planning (35–37). However, such drive toward a desired dose to OARs for TMI in this study was well under control with KBP as indicated by the improved plan qualities reported in **Table 3**. Note that the slight increase in total plan MU, a common indicator of plan complexity, was statistically insignificant.

Another important value of the knowledge-based approach is the prospect of plan quality management. KBP resulted in comparable or better dosimetric parameters mostly as shown in **Table 2** and **Table 3**. Furthermore, significantly narrower distributions were produced for the mean dose to lungs and PTVs, as well as for homogeneity and conformity indices. These are strong indications that the plan quality adheres to a set of criteria defined by the transferable knowledge and expertise. This could potentially enable widespread clinical application of TMI. In fact, our study design itself had the training from 51 plans in one clinic achieve treatment planning of 22 new patients in another clinic. It demonstrates that the knowledge-based artificial intelligence approach can facilitate multi-center clinical trials of TMI with automation and data sharing as foreseen by Wong et al. (38).

## 5 Conclusions

A knowledge-based DVH estimation model was successfully configured for linac-based multiple-isocenter TMI planning and was used to generate plans for test patients from another clinic with plan quality equivalent or superior compared to the references. VMAT-TMI powered by KBP can potentially support uniform dosimetric quality among different users and clinics, and can reduce treatment planning time and effort by providing a goal attainable within a few iterative optimizations allowing widespread clinical use of TMI.

## Data availability statement

The original contributions presented in the study are included in the article/supplementary material. Further inquiries can be directed to the corresponding author.

## Ethics statement

The studies involving human participants were reviewed and approved by Institutional Review Boards of University of Chicago Medicine and University of Illinois at Chicago. The patients/participants provided their written informed consent to participate in this study.

## Author contributions

KA wrote the first draft of the manuscript. BA contributed to manuscript revision. All authors contributed to the article and approved the submitted version.

## Conflict of interest

The authors declare that the research was conducted in the absence of any commercial or financial relationships that could be construed as a potential conflict of interest.

## Publisher’s note

All claims expressed in this article are solely those of the authors and do not necessarily represent those of their affiliated organizations, or those of the publisher, the editors and the reviewers. Any product that may be evaluated in this article, or claim that may be made by its manufacturer, is not guaranteed or endorsed by the publisher.
