# Supplementary material for: Knowledge-based planning for multi-isocenter VMAT total marrow irradiation
Source: Front Oncol. 2022 Oct 4;12:942685. doi: 10.3389/fonc.2022.942685 (PMC9577613; doi:10.3389/fonc.2022.942685)
Supplement: Supplementary file 1 [file DataSheet_1.pdf]

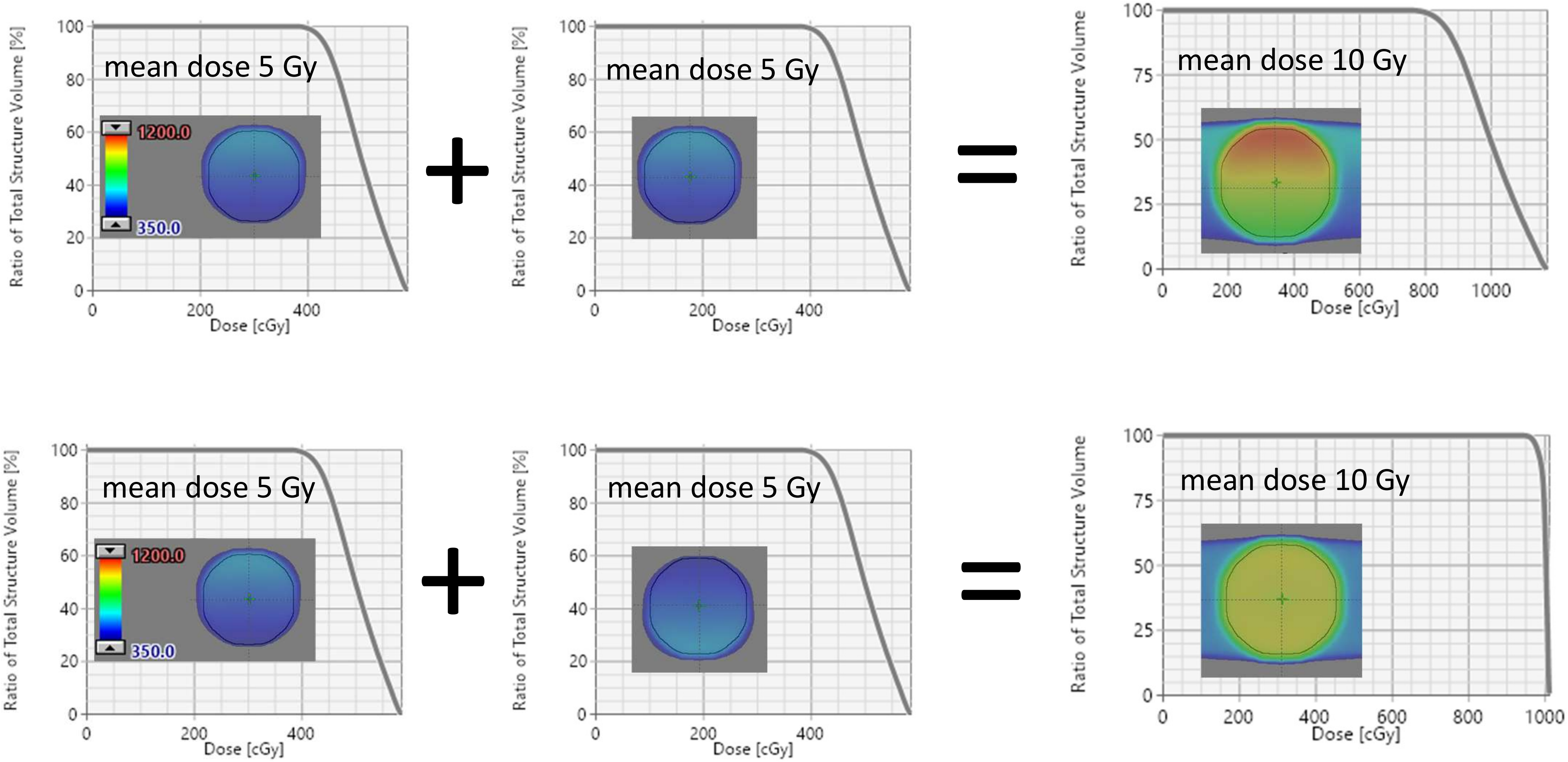

DVH does not include spatial information. Identical subplan DVHs can result in totally different composite DVHs.
